# Exploring Genome-Wide Mutation Dynamics and Bacterial Cellulose Impairment in *Komagataeibacter intermedius* Cultivated Under Agitation Stress

**DOI:** 10.34133/csbj.0009

**Published:** 2026-03-18

**Authors:** Davide Bersanetti, Rahul Mangayil

**Affiliations:** Department of Bioproducts and Biosystems, Aalto University, Espoo, Finland.

## Abstract

**Background:** Bacterial cellulose (BC), natively synthesized by *Komagataeibacter* spp., is a biodegradable biomaterial with superior mechanical properties. However, under agitated cultivation, cellulose-producing strains (Cel^+^) often transition to nonproducing mutants (Cel^−^), restricting scalability and hindering widespread use. Agitation-associated shear stress, elevated oxygen levels, and genetic mutations have been linked to the emergence of the Cel^−^ phenotype. A genome-wide investigation that considers population heterogeneity and dynamics is essential to reveal the mutational landscape and evolutionary processes driving this phenotypic shift. **Results:** Over successive rounds of agitated cultivation, *K. intermedius* ENS15 transitioned to a planktonic state, losing BC production. Whole-genome sequencing revealed both structural variations (SVs) and nonstructural variations (NSVs). Contrary to the previous reports, SVs, including insertion sequence-mediated junction events, did not affect the genes related to BC synthesis. Instead, the accumulation and positive selection of NSVs, such as frameshift and replication slippage events in key BC-related genes, strongly correlated with the loss of cellulose synthesis. **Conclusions:** This study provides the first genome-wide population-level analysis revealing mutational dynamics underlying the BC phenotypic switch in *Komagataeibacter* spp. cultivated under agitated conditions. We show that the BC-related gene mutations are not solely driven by SVs, with NSVs emerging as equally critical contributors. Furthermore, genomic evidence suggests possible involvement of regulatory mechanisms, such as quorum sensing and cyclic dimeric guanosine monophosphate signaling, prompting future studies on regulatory processes under agitated cultivation conditions. These findings highlight the importance of examining genetic heterogeneity to understand phenotypic adaptation for strain improvement strategies.

## Graphical Abstract

**Figure:**
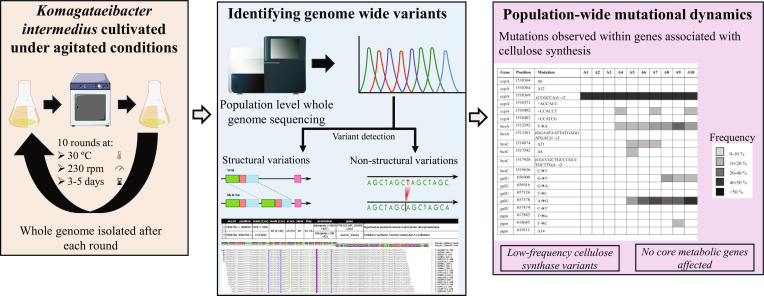

## Background

Bacterial cellulose (BC), primarily produced by *Komagataeibacter* spp. (formerly *Acetobacter* and *Gluconacetobacter*), has attracted growing interest in wide-ranging applications. Unlike plant-derived cellulose, high purity (devoid of lignin, hemicellulose, and pectin), superior physical and mechanical properties, along with the nontoxic and biodegradable nature have established BC as a sustainable biomaterial [1].

The BC synthesis machinery, encoded by the bacterial cellulose synthase (bcs) operon, comprises the core genes *bcsA*, *bcsB*, *bcsC*, and *bcsD* and accessory genes such as *bcsZ* (endoglucanase, also referred to as *CMCax*), *ccpAx* (cellulose complementing factor A), and *bglX* (beta-glucosidase) [2]. Briefly, uridine diphosphate glucose (UDP-glucose), synthesized through the catalytic activities of phosphoglucomutase (*pgm*, converting glucose-6-phosphate to glucose-1-phosphate) and uridine triphosphate-glucose-1-phosphate uridylyltransferase (*galU* converting glucose-1-phosphate to UDP-glucose), serves as the substrate for cyclic dimeric guanosine monophosphate (c-di-GMP)-activated bcs operon that polymerizes the glucosyl residues to β-1,4-glucan chains [2,3]. Although *Komagataeibacter* spp. typically harbor multiple *bcs* operons (~4 to 5 genome copies), only 1 constitutes a complete operon, referred to as *bcsABCD* [4]. A second operon, containing *bcsABC*, *bcsX* (putative SGNH/GDSL hydrolase), *bcsY* (putative acyltransferase) genes, has been associated with the synthesis of amorphous or acylated cellulose and has been recently referred to as *bcsAB2XYC2* [4], while the remaining operons generally encode only *bcsAB* or *bcsABC* genes [5,6].

Despite the advancements in *Komagataeibacter* bioprocessing, scale-up of BC production remains constrained by the spontaneous emergence of noncellulose-producing (Cel^−^) mutants under agitated cultivation conditions [7]. Conventionally, *Komagataeibacter* spp. are cultivated under static conditions. Being an obligate aerobe, the cells migrate along the dissolved oxygen gradient toward air–liquid interface producing BC pellicles [8]. Prolonged synthesis at this interface hinders oxygen diffusion, leading to a hypoxic environment [9] for the cells confined within the BC pellicle. To overcome this limitation, conventional shake flasks and bioreactors have been employed to enhance oxygen mass transfer, for improved productivity [10]. However, agitated conditions often generate random Cel^−^ mutants, impacting BC production [11–13]. Although the molecular basis remains unclear, shear stress and elevated oxygen in agitated cultures have been proposed as possible drivers of this phenotypic switch [7,11,13]. One hypothesis is that unlike in static conditions where the pellicle facilitates oxygen availability for cell growth at the air–liquid interface, BC synthesis during agitated cultivation provides no selective advantage [11]. Although limited, studies in *Komagataeibacter* spp. show that elevated oxygen levels reduce *pgm* and *galU* (also referred as *gtaB*) [14] expression and increases phosphodiesterase (PDE) activity, resulting in lower cellular c-di-GMP levels [15], a central regulator for motility, stress responses, virulence, and biofilm formation [16].

Appearance of Cel^−^ mutants under shaking conditions has also been linked to both insertion sequence (IS)-mediated structural variations (SVs) and nonstructural variations (NSVs) disrupting the BC-related genes [11]. Coucheron [17] reported an IS1031 disrupting the bcs operon of *K. hansenii* 23769 under agitated cultivation, while Hur et al. [18] observed a similar IS1031 insertion within the *bcsA* gene of *K. xylinus* DSM2325 under the same conditions. Furthermore, Krystynowicz et al. [13] demonstrated that in Cel^−^ *A. xylinum* E25, a single-base (T) deletion impaired both pgm and galU activity, thereby UDP-glucose synthesis.

This study aims to improve our understanding of the mechanisms underlying the emergence of Cel^−^ mutants during agitated cultivations by applying population-level sequencing to characterize genome-wide mutational landscapes in heterogeneous cell populations. Traditionally, insights into Cel^−^ mutations have been drawn from single-colony isolates [13,17,18], obtained after shaking cultivations, focusing largely on genes involved in cellulose synthesis. While informative, these studies have tended to overlook population complexity and lack a comprehensive genome-wide perspective, limiting the identification of additional mutational and adaptive drivers. Our approach moves beyond these limitations by providing a more holistic view of mutational dynamics and evolutionary processes during agitated cultivation. By employing population-level sequencing, we capture the diverse genomic variations within agitated cultivations, providing new insights into the Cel^−^ mutant phenomenon. Furthermore, by integrating population genomics with a careful review of regulatory pathways responsive to environmental changes, we propose mechanistic interpretations linking cultivation conditions to the observed genetic patterns, which warrant further investigation through dedicated studies.

## Results

### BC production during agitated cultivation

*K. intermedius* ENS15 (K.ENS15) was cultivated under agitated conditions in 3 parallel cultivations (Flasks A, B, and C) for 10 successive rounds of 3 to 5 d each (see Methods). After the first round, BC production was evident as a single agglomerated pellicle, and the culture medium remained clear with no noticeable planktonic growth (Additional File 1: Fig. S1). By the second round, BC production persisted in all cultivation lines but was accompanied by visible planktonic growth. A table summarizing the onset of planktonic growth and complete loss of BC production across the flasks (A, B, and C) is provided in the additional files (Additional File 1: Table S1). To evaluate the cell growth across the cultivation rounds, we measured optical density at 600 nm (OD_600nm_) at the end of each round from both lysed BC pellicles and planktonic cultures. In Flask A, OD_600nm_ values ranged between 1.1 and 3.3, corresponding to an average of 4.7 generations per round. Flask B showed OD_600nm_ values between 1.1 and 2.6, averaging 4.5 generations per round. Similarly, Flask C exhibited OD_600nm_ values between 1.1 and 2.6, with an average of 4.7 generations per round (Additional File 1: Fig. S2). Overall, all the flasks showed impaired BC production before the end of the experiment, without growth impairment.

### Static cultivation to assess Cel^−^ to Cel^+^ reversion

Ability of Cel^−^ mutants to revert to Cel^+^ phenotype has been reported in literature [11]. To assess the reversion capacity, we recultivated independently the cells from each agitated cultivation round under static conditions, alongside the Cel^+^ parental strain. In contrast to the Cel^+^ parental strain (0.9 g·l^−1^ BC), agitated cultures showed lower or comparable titers with occasional spikes: Flask A ranged from 1.1 to 0.1 g·l^−1^, Flask B from 1.3 to 0.1 g·l^−1^, and Flask C from 1.3 to 0.3 g·l^−1^ (Additional File 1: Fig. S3).

### Population-level detection of IS-mediated SVs and junction events

**Fig. 1.**
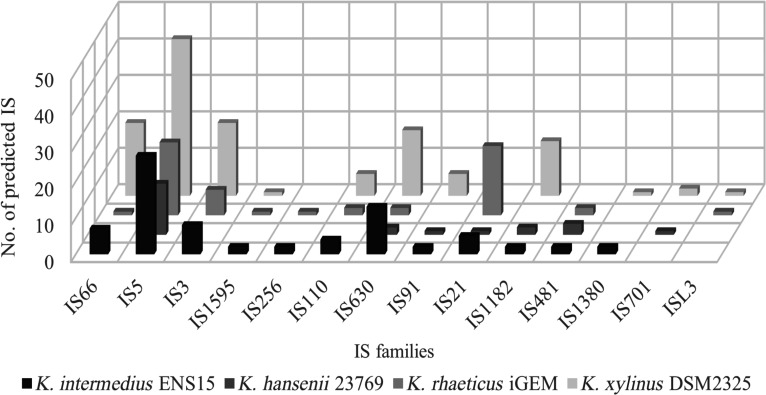
ISEScan predicted distribution of IS families in *K. intermedius* ENS15, *K. hansenii* 23769, *K. rhaeticus* iGEM, and *K. xylinus* DSM2325.

SVs mediated by ISs and associated deletions have previously been reported in *Komagataeibacter* cells grown under agitated conditions [6,17,18]. Prior analysis of the K.ENS15 reference genome (National Center for Biotechnology Information accession number, GCA_021555195.1) identified 73 IS elements. Figure 1 presents the IS identified in K.ENS15 by ISEScan, alongside those found in other characterized *Komagataeibacter* spp. A complete list of IS elements in K.ENS15, including genomic locations, is available in Additional File 2.

The unprocessed Breseq outputs are provided in Additional File 3, along with manually curated tables comparing the junction (JC) events between the parental strain and cells from the 3 flasks (Additional File 4). Majority of SVs detected by Breseq (a total of 139 events) were categorized as “unassigned junction events”. JC events represent instances where 2 genomic regions, typically distant in the reference genome, appear adjacent in sequencing reads. These events frequently arise from IS activity leading to insertions, deletions, or duplications. The “unassigned” designation indicates cases where the JC cannot be precisely resolved, often because it spans regions exceeding the sequencing read length or involves repetitive sequences.

**Fig. 2.**
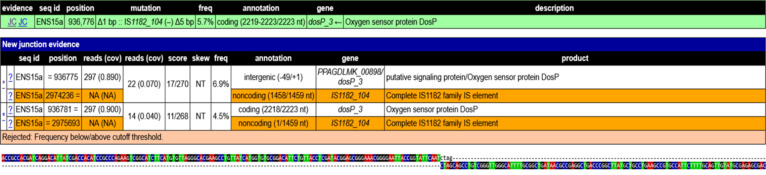
Breseq evidence of a JC event between IS1182_104 and the intergenic region between a putative signaling protein and an oxygen sensor protein. Rows corresponding to IS1182 are highlighted in orange. In figure labels: “Position” refers to the genomic position of the interested sequence, the first “reads (cov)” show the amount of reads and the relative coverage of the sequenced region, the second “reads (cov)” report the number of reads and the coverage supporting the mutation, and “freq” denotes the percentage of reads containing the variant.

Analysis revealed that most of the JC events were inherited from the Cel^+^ parental strain and persisted across the agitated cultivations (Additional File 4). However, manual curation identified several JC events unique to the agitated cultures, involving an IS element and a non-IS genomic region (Additional File 4). One example is a unique JC event detected after Round 10 in Flask C (frequency 5.7%, coverage 0.055), involving the IS1182_104 element (genomic position 2974235 to 2975693 bp) and an intergenic region of a gene encoding putative signaling protein and an oxygen-sensing gene (*dosp3*) (genomic position 936726 to 936776 bp) as shown in Fig. 2. Basic Local Alignment Search Tool (BLAST) [19] analysis of the putative signaling protein identified it as a putative diguanylate cyclase (DGC)/PDE domain-containing protein involved in c-di-GMP signaling.

Previous studies have reported IS-mediated mutations to affect BC-related genes during agitated cultivation [17,18]. Such loci were specifically examined in our dataset. The only IS-related event directly involving a BC gene was already present in the Cel^+^ parental strain and was inherited through the shaken cultivations (Additional Files 3 and 4). This event, supported by 2 JC events, was observed in the *bcsAB2XYC2* operon [4], originally referred to as bcs operon IV by Cannazza et al. [20], between the *bcsABII* (genomic position 3299830 to 3304158 bp) and an upstream putative acetyl-coenzyme A synthase (genomic position 3304224 to 3306005 bp) (Additional File 1: Figs. S4 and S5). Comparative analyses suggest that this operon resembles those implicated in amorphous cellulose synthesis [6]. In the parental strain, the 2 JC events were present with frequencies of 14.1% and 28.7%. In the shaken cultures, these frequencies fluctuated between 11.5%–21.2% and 23.4%–35.9% in Flask A; 10.0%–19.1% and 22.5%–36.4% in Flask B; and 12.2%–22.6% and 24.3%–36.2% in Flask C (Additional Files 3 and 4). Together, these results indicate that, contrary to previous reports, a direct association between IS-mediated events and the Cel^+^-to-Cel^−^ transition is not conserved across *Komagataeibacter* spp., while also providing a clear image of how IS activity affects the whole genome of K.ENS15, rising concerns on genome stability.

### Population-level detection of NSVs

Consistent with the JC event observations, the majority of the NSV events (a total of 2,138 detected) were inherited from the Cel^+^ parental strain. Nevertheless, mutations unique to the shaken cultivations were also identified. A comprehensive comparison of all NSVs detected in both the parental strain and shaken cultures, along with “unassigned” JC events are presented in Additional File 5. Most NSVs unique to the agitated cultivations showed no evidence of positive selection over time; instead. they appeared after specific cultivation rounds and were not retained long-term. For instance, in Flasks A and C, single-nucleotide polymorphisms (SNPs) were detected in *ydaD_1* encoding general stress protein 39 (genomic position 2297035 to 2297895 bp). In Flask A, after Round 1, a G➔A substitution at genomic position 2297613 was observed at 10.4% frequency, causing an R95W amino acid change. A similar SNP appeared in Flask C after Round 7 at a frequency of 9.3%. Another G➔A substitution at genomic position 2297622 bp, detected in Flask A after Round 8, did not cause an amino acid change. In Flask C, an SNP cause an amino acid substitution (R90C) after Round 7 at a frequency of 7.2%. The analysis of detected NSVs showed that no core metabolic genes were affected, indicating that the agitated culture per se is not harmful to the growth and survival of *Komagataeibacter* ENS15, proved by the persistence of growth throughout the experiment.

#### NSV events within key genes involved in BC synthesis

As anticipated based on prior studies, NSV events were detected in several BC-related key genes, including *ccpA*, *bcsAI*, *bcsCI*, *galU*, and *pgm* (Table 1). Several of these mutations, absent in Cel^+^ parental cells, displayed evidence of positive selection over time with some recurring independently across all 3 flasks.

**Table 1.** NSV events within key genes involved in BC-synthesis ^a^

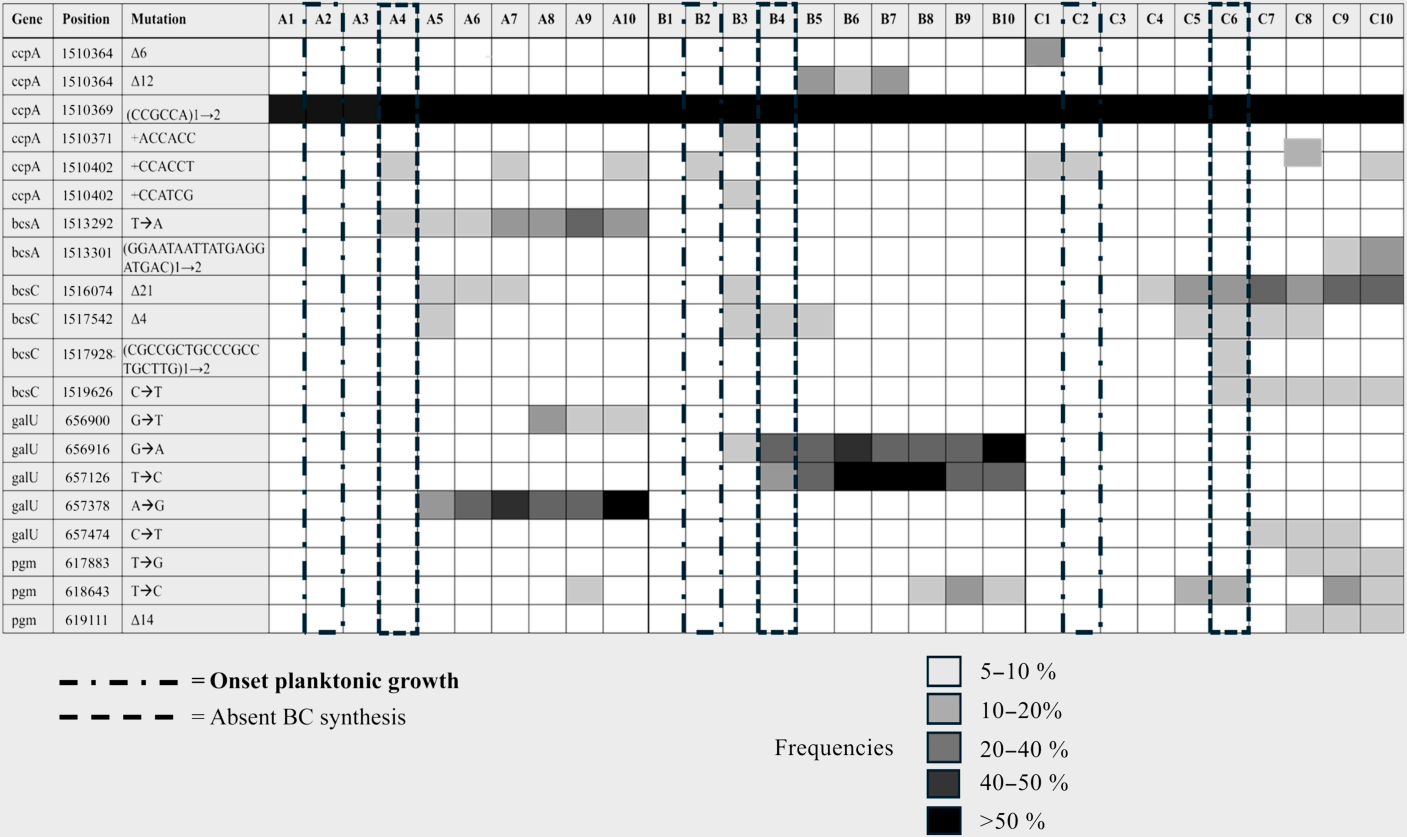

^a^ The table is a summary report derived from Additional File 3. The table depicts color-coded values of the frequency of the mutations affecting genes involved in BC synthesis highlighting the positive selection of certain mutations. The table additionally provides the genomic position of such mutations.

##### NSV events in ccpA

Notably, most NSVs were clustered within the accessory gene *ccpA*, specifically within a hotspot region (genomic position 1510364 to 1510402 bp) encoding 13 consecutive proline residues derived from repeated CCG/CCA triplets prone to replication slippage (Additional File 1: Fig. S6) [21]. A parental slippage event, expanding 1 to 2 copies of the CCGCCA repeat, was retained at high frequency (83% to 88%) across all rounds and flasks (Table 1). Additional lineage-specific insertions also arose within this hotspot. In Flask A, a +CCACCT insertion (adding 2 prolines) appeared after Rounds 4, 7, and 10 with frequencies ranging from 5.6% to 7.8%. In Flask B, the same insertion was detected after Round 2, alongside 2 further insertions, +ACCACC and +CCATCG introducing 2 threonine and a proline–serine pair, respectively, present with similar frequency values. Flask C showed a heterogeneous population carrying the +CCACCT at Rounds 1 and 2, disappearing in intermediate rounds, and reemerging at Round 10 (frequences ranging between 5.0% and 8.9%). Additionally, small deletions of 6 to 12 bp, removing 2 to 4 proline residues (genomic position 1510364 to 1510369/1510375 bp), appeared transiently in Flasks B and C at frequencies between 12% and 18% (between Rounds 5 and 7 in Flask B and after Round 1 in Flask C; Table 1).

##### NSV affecting bcsA

Within the *bcsABCD* operon, NSVs were detected exclusively in *bcsA* and *bcsC*. In *bcsA*, a nonsense mutation (TAT → TAA) introducing a premature stop codon (genomic position 1513292 bp) emerged in Flask A. This mutation, absent in Flasks B and C, was gradually enriched over the course of cultivation, increasing in frequency from 9.4% at Round 4, when a clear absence of cellulose production was first observed, to 15% to 36% by Rounds 9 and 10. In Flask C, a 20-bp duplication (GGAATAATTATGAGGATGAC, genomic position 1513282 to 1513301 bp) in *bcsA*, causing a frameshift and the emergence of 2 stop codons (Additional File 1: Fig. S7), was detected during the final 2 cultivation rounds (Table 1). Overall, *bcsA* variants were detected only in Flasks A and C, both predicted to disrupt the entire BC synthase complex.

##### NSV affecting bcsC

In contrast to *bcsA*, the *bcsC* gene was affected across all flasks. Two recurrent mutations were identified: a 21-bp deletion (GCACGTTTCTGGTTGCAGCAG, genomic position 1516074 to 1516094 bp) and a 4-bp deletion (GGCA, genomic position 1517542 to 1517545 bp). The 21-bp deletion removed the amino acid sequence ARFWLQQ within a tetratricopeptide repeat region of *bcsC*. Using the SwissModel tool [22], we created a 3-dimensional model of the mutated *bcsC*, to compare it against the wild type *bcsC* (Additional File 1: Fig. S8). The 4-bp deletion caused a frameshift and introduced premature stop codons (Additional File 1: Fig. S9). Although the timing of these deletions varied across the flasks, the 21-bp deletion was positively selected only in Flask C. This deletion, initially detected with a frequency of 5.7% at Round 4, before the disruption of cellulose production observed at Round 5 (Additional File 1: Fig. S1), increased in frequency, reaching 35% by Round 10. In contrast, the 4-bp deletion remained at low frequencies (6% to 10%) and showed no evidence of positive selection (Table 1). Additional events were also identified in Flask C. A duplication event, causing a frameshift and the introduction of multiple stop codons (CGCCGCTGCCCGCCTGCTTG_1➔2_, genomic position 1517909 to 1517928 bp), was present after Round 6 (Additional File 1: Fig. S10). In the same round, an SNP (CAG→TAG, genomic position 1519626 bp) introduced a premature stop codon and persisted at low frequencies (6% to 9%) within the heterogeneous population through Round 10, coinciding with the rounds characterized by complete loss of cellulose production in Flask C (Additional File 1: Fig. S1).

##### NSV affecting galU

NSV events were identified in *galU*, encoding uridine triphosphate-glucose-1-phosphate uridylyltransferase, essential for UDP-glucose formation and BC synthesis. These mutations appeared across all shaken cultivations, with varying type and temporal dynamics (Table 1). For instance, in Flask A, an A→G substitution resulting in a tyrosine-to-cysteine amino acid change at position 172 (Y172C) emerged at Round 5, 1 round after complete loss of cellulose production was first observed. This variant was initially detected at a frequency of 19% and subsequently increased in frequency, reaching 75% by Round 10. A second mutation causing an alanine-to-serine substitution at position 13 (A13S) appeared at Round 8 and retained until Round 10. Flask B showed 2 additional point mutations absent in Flask A. One of these was a G→A substitution causing an arginine-to-histidine change at position 18 (R18H), which was detected at Round 3, the first round in which cellulose production appeared compromised, at a frequency of 8%. This mutation was positively selected, reaching a frequency of 72% by Round 10. Additionally, a T→C substitution causing a leucine-to-proline change at position 88 (L88P) appeared at Round 4, where cellulose production was completely absent, with a frequency of 11.8%. Subsequently, it reached a frequency of 63% at Round 7 before declining and stabilizing at 23% by Round 10. Although similar mutations were absent from heterogeneous populations in Flask C, a nonsynonymous SNP (S204F) was detected between Rounds 7 and 9, which was absent by Round 10.

##### NSV affecting pgm

NSV events were identified in the *pgm* gene, which encodes phosphoglucomutase, the enzyme catalyzing the conversion of glucose-6-phosphate to glucose-1-phosphate, a key step in UDP-glucose biosynthesis. Mutations in *pgm* were observed across all shaken cultivations with distinct frequencies and patterns (Table 1). Three key NSV events were detected: 2 point mutations and 1 deletion. Among these, a T➔C substitution causing a lysine-to-glutamate amino acid change at position 158 (K158E) was present in Flasks A, B, and C at low frequencies ranging from 5% to 16%. The other 2 SNV variants, a T➔G mutation resulting in an aspartate-to-alanine change at position 411 (D411A) and a 14-bp deletion (GCATTGACTGCTCC, bp 619111 to 619124) that removes the start codon, were identified exclusively in Flask C from Round 8 onward (Additional File 1: Fig. S11). Initially, the frequencies of the deletion and mutation were 10.4% and 8.4%, respectively, and they persisted until the final cultivation round, with final frequency values of 9.2% and 18.5% (Additional File 5).

## Discussion

Our empirical observations during shaking cultivations confirmed a progressive reduction in BC production, accompanied by increased planktonic cell growth, consistent with previous studies [23,24]. This phenomenon has been linked to mutations identified in homogeneous population isolated as Cel^−^ mutants on agar plates [13,18,25].

Unlike the conventional approaches that isolate single colonies and detect mutations at 100% frequency, our study uniquely analyzed mutations from the perspective of a natural heterogeneous cell population, preserving the broader genetic diversity context. The persistence of multiple mutations at intermediate frequencies across cultivation rounds, as visualized in Table 1, argues against selective sweeps and instead suggests the coexistence of subpopulations under heterogeneous or fluctuating selection pressures during agitated cultivation.

The BC synthesis genes were affected across all 3 flasks, with some mutations undergoing clear positive selection over time, indicating potential growth advantages. Notably, no mutations were detected within genes involved in core metabolic pathways.

NSVs in key BC synthesis genes were consistent with previous findings (Table 1) [26]. However, our population-based approach revealed that the mutations associated with impaired or absent BC production were not predominant within the populations and, in some cases, were not detected. Furthermore, each flask exhibited distinct mutational landscapes, yet BC production was ultimately suppressed in all, independent of the predominant genotypes or the presence of specific subpopulations. Together, these observations suggest that additional mechanisms, acting broadly across flasks, may have contributed to the suppression of BC synthesis under agitated conditions.

Recently, Zhang et al. [27] reported *K. xylinus* 2955 to possess a complex c-di-GMP regulatory mechanism, comprising 10 genes with tandem PDE/DGC activity (GGDEF−EAL domain proteins). The study established that the knockout of *pde*, encoding PDE that regulates the degradation of total cellular c-di-GMP, led to a 48% increment in BC synthesis. Complementary findings by Tuckerman et al.in *Escherichia coli* demonstrated a positive correlation of elevated oxygen levels to enhanced PDE activity, while Huang et al. reported that increased oxygen concentrations led to a down-regulation of the BC pathway gene expression in *K. xylinus* CGMCC 2955 [14,15]. Thes studies, when critically analyzed together, support a scenario where an increment of oxygen levels directly affects BC production via a modulation of regulatory pathways and gene expression levels.

Coherently, our BLAST analysis identified 9 loci encoding putative PDE/DGC activity in K.ENS15. Cannazza et al. [20] originally annotated 7 of these as oxygen-sensing sensor proteins (*dosP*), indicating a possible active modulation of c-di-GMP metabolism in response to oxygen (Additional File 1: Table S2). No mutations affecting these genes were observed within the 3 shaken cultures, except for a late-round mutation in *dosP_3* in Flask C which appeared too late to influence BC production (see the “Population-level detection of insertion sequence-mediated SVs and junction events” section).

In agitated cultures, dissolved oxygen levels are higher compared to static conditions [28]. Based on our findings and the discussed literature [14,15,27], we suggest that increased oxygen availability may influence regulatory mechanisms associated with the transition from Cel^+^ to Cel^−^ phenotype. Such modulation could enhance PDE activity and reduce overall cellular c-di-GMP levels, thereby creating conditions that disfavor cellulose synthesis. Under these conditions, subpopulations carrying genotypes incompatible with BC production may become enriched, consistent with observations reported in previous studies [7,13].

This hypothesis is further supported by the reversibility from the Cel^−^ to Cel^+^ phenotype under static cultivation, which correlates to the changes in oxygen availability [24]. To explore this possibility, we performed a preliminary experiment to quantify the total intracellular c-di-GMP levels in cells from Rounds 1 and 10 of Flask A lineage [29]. Preliminary fluorometric measurements indicated a 28% ± 4% decrease in total intracellular c-di-GMP (Additional File 1: Fig. S12). While these data support the hypothesis, global c-di-GMP levels may not fully reflect the localized pools that are critical for BC regulation [27].

Other regulatory mechanisms have been identified as modulator of BC synthesis. *N*-acyl homoserine lactone quorum sensing (QS) regulation of PDE activity via alternative signaling routes has been reported in *Komagataeibacter* spp. [30,31]. Additionally, Valera et al. [32] demonstrated quorum quenching (QQ) activity in *K. europaeus* via the *N*-acyl homoserine lactone-degrading GqqA protein (also present in K.ENS15, genomic position 635676 to 636521 bp), which halted BC production [32].

Additionally, despite meticulous handling (static cultivation for BC synthesis, minimal-agitation BC lysis, and cryopreservation for glycerol stock preparation), the presence of IS insertions and mutations in the parental strain indicates genomic divergence from the originally isolated strain [20] and the coexistence of several subpopulations within what can now be considered as the parental stock. This underscores the inherent genomic instability of *Komagataeibacter* spp. and emphasizes the need for rigorous sequence verifications when working with these bacteria. At the same time, despite the presence of multiple subpopulations, the parental stock remained an active cellulose producer. Our analysis therefore identifies candidate genes that, although mutated across subpopulations under the studied conditions, did not impair BC synthesis, offering possible targets for future engineering efforts.

Future research will prioritize the characterization of both localized and global c-di-GMP pools in *Komagataeibacter* spp., together with transcriptomic and metabolomic analyses under controlled oxygen conditions. Such approaches will help clarify the role of the c-di-GMP regulation, QS, and QQ and may reveal additional regulatory mechanisms influencing BC production under agitated or industrially relevant cultivation conditions. Alongside these studies, we will expand our approach to additional strains and glycerol stocks, utilizing long-read sequencing approaches to enhance the resolution of IS events and define the genome evolution of *Komagataeibacter* spp. over the years despite meticulous maintenance.

## Conclusion

This study presents the first comprehensive characterization of the mutational landscape within a heterogeneous population of *Komagataeibacter* spp. undergoing the well-documented Cel^+^-to-Cel^−^ phenotypic switch under agitated cultivation. Our findings suggest that the loss BC production might not be driven exclusively by mutations in BC biosynthetic genes but is likely influenced by additional regulatory mechanisms. In this context, regulatory pathways involving c-di-GMP signaling, as well as QS and QQ, emerge as important contributing factors, which will be subject to future dedicated studies to characterize their involvement in the widely observed Cel^+^-to-Cel^−^ phenotypic switch under agitated cultivation.

## Materials and Methods

### Chemicals and materials

GeneJet genomic DNA purification Kit was acquired from Fisher Scientific (Vantaa, Finland). Citric acid, glucose, sodium chloride, and sodium hydroxide were purchased from VWR International Oy (Helsinki, Finland). Cellulase from *Trichoderma reesei*, disodium hydrogen phosphate, potassium dihydrogen phosphate, and potassium chloride were acquired from Merck (Espoo, Finland). Yeast extract and bacteriological peptone were acquired from Neogen (Lansing, USA). The cyclic-di-GMP Assay kit was purchased from Lucerna (New York, USA). Black, flat-bottomed 96-well plates were purchased from VWR International Oy (Helsinki, Finland).

### Strain, media, and agitated cultivation

K.ENS15 was isolated by Cannazza et al. [20]. For preculture preparation, 100 μl of glycerol stock (parental strain) was inoculated in 10 ml of Hestrin–Schramm (HS)-glucose medium [20] and incubated under static conditions for 5 d at 30 °C. The synthesized BC was lysed overnight using 2% cellulase to release the entrapped cells, which were subsequently washed thrice with sterile 1× phosphate-buffered saline (PBS) [20].

The washed cells were used as inoculum for agitated cultivations (initial OD_600nm_ of 0.08), conducted in 250-ml Erlenmeyer flasks, with 3 parallel cultivation lines designated as Flask A, Flask B, and Flask C. Cultivations were conducted in 50 ml of HS-glucose media at 30 °C and 230 rpm for 10 consecutive rounds, each lasting 3 to 5 d [18]. At the end of each round, 1 ml of cellulase was added irrespective of observable cellulose formation [20] After the PBS washes, the ODs of the resulting heterogeneous cell suspensions were measured and used both as inoculum for the following round of agitated and static cultivation and as a source for sequencing. A flask with containing HS-glucose media was included in each round as a contamination control.

### Static cultivations and BC processing

Static cultivation experiments were performed using a single biological replicate per round and lineage in 50-ml tubes containing 10 ml of HS-glucose medium inoculated to an initial OD of 0.08. Cells were harvested from agitated cultivation rounds following cellulase treatment and PBS washing. Cultures were incubated for 4 d at 30 °C, and BC pellicles were processed as described by Cannazza et al. [33].

### Genome sequencing, variant calling, and bioinformatics

Genomic DNA was extracted using the GeneJet Genomic DNA Purification Kit as per manufacturer’s instructions (Fisher Scientific, Finland). One sample per cultivation round and flask was subjected to paired-end sequencing (Illumina NovaSeq X Plus; Novogene Gmbh, Planegg, Germany).

The sequencing was performed using 150-bp read, resulting in an insert size of 320 to 350 bp. The reads quality was assessed using FastQC (v0.12.1) [34] and trimmed using Trimmomatic (v1.39) [35] with the following settings (Minlen – 50, Leading – 3, Trailing – 3, Sliding Window – 4:20). The achieved coverage depth, measured using Samtools depth [36] in default setting, is >120× for all the samples.

Genetic variation analysis was then conducted using Breseq (v0.39.0) [37–41] in polymorphism mode with default settings (with a minimum frequency threshold at 5%), using the deposited K.ENS15 reference sequence (GenBank accession: GCA_021555195.1). Prior to variant calling, ISs in the reference genome were annotated using ISEScan (v.1.7.2.2) [42]. This step proved to be essential since the widely used ISaga [43] was unavailable (accessed 2024 to 2025). Detailed instructions for integrating ISEScan with Breseq are described in the Breseq user manual [39]. Genomic samples analyzed included those collected at the end of each cultivation round as well as the parental strain. To compare the mutational landscapes, raw Breseq outputs were further processed using GDTools [39] following the Breseq manual. Only NSV and the JC events not classified as “unassigned” were included in GDTools-based comparisons (Additional File 5). The JC events classified as “unassigned junction events” by Breseq were instead separated into manually curated tables. Manual curation was based solely on annotation of the genomic regions involved and was performed to facilitate downstream analysis of the dataset. This curation resulted in 3 macro groups: (a) JC events involving an annotated IS and another genomic region (reported in Additional File 4); (b) JC events involving 2 nonannotated regions; and (c) JC events involving a nonannotated region and an annotated region not classified as an IS. Events belonging to categories (b) and (c) are provided as part of the raw Breseq output (Additional File 3).

The bioinformatics analyses were performed on the Puhti supercomputer provided by CSC–IT Center for Science (Finland) [44]. The BLAST provided by National Center for Biotechnology Information [19] was utilized to examine sequences of interest and manually annotate hypothetical proteins. Geneious Prime 2025.0.3 [45] was used to visualize the effect of the NSV within key genes involved in BC synthesis, generating Figs. S6, S7, and S9 to S11 displayed in Additional File 1.

### C-di-GMP fluorometric assay

The c-di-GMP fluorometric assay was conducted according to supplier’s instructions, using black, flat-bottomed 96-well plates with 1 modification. To enhance accessibility of intracellular c-di-GMP, cell pellets obtained from PBS-washed cultures at the end of the shaking cycles were briefly sonicated at 20% amplitude, after resuspension in 4 ml of 1× PBS [29]. The assay was conducted using 1 biological sample per round, analyzed in 3 technical replicates. Fluorescence measurements were conducted using a Biotek Cytation 3 with emission at 485 nm and excitation at 528 nm. Background fluorescence was subtracted from recorded values and normalized by OD_600nm_ values of the resuspended pellets. A parallel negative control, in which all the reagents were added except for the c-di-GMP sensor molecule, confirmed that the detected fluorescence signal originated from intracellular c-di-GMP.

## Data Availability

All the raw data, except the c-di-GMP data, are included in the additional files. The c-di-GMP raw data are available upon request.
